# Attenuated lipopolysaccharide-induced inflammatory bladder hypersensitivity in mice deficient of transient receptor potential ankilin1

**DOI:** 10.1038/s41598-018-33967-x

**Published:** 2018-10-23

**Authors:** Jun Kamei, Naoki Aizawa, Takayuki Nakagawa, Shuji Kaneko, Haruki Kume, Yukio Homma, Yasuhiko Igawa

**Affiliations:** 1 Department of Continence Medicine, The University of Tokyo Graduate School of Medicine, Tokyo, Japan; 2 Department of Urology, The University of Tokyo Graduate School of Medicine, Tokyo, Japan; 3 Department of Clinical Pharmacology and Therapeutics, Kyoto University Hospital, Kyoto, Japan; 4 Department of Molecular Pharmacology, Graduate School of Pharmaceutical Sciences, Kyoto University, Kyoto, Japan; 5 Department of Urology, Japan Red Cross Medical Center, Tokyo, Japan

## Abstract

Transient receptor potential ankyrin 1 (TRPA1) channel expressed by urothelial cells and bladder sensory nerve fibers might act as a bladder mechanosensor and nociceptive transducer. To disclose the role of TRPA1 in bladder function and inflammation-associated hypersensitivity, we evaluated *in vitro* and *in vivo* bladder function and inflammatory mechanosensory and nociceptive responses to intravesical lipopolysaccharide (LPS)-instillation in wild type (WT) and TRPA1-knock out (KO) mice. At baseline before treatment, no significant differences were observed in frequency volume variables, *in vitro* detrusor contractility, and cystometric parameters between the two groups in either sex. LPS-instillation significantly increased voiding frequency and decreased mean voided volume at 24–48 hours after instillation in WT but not in TRPA1-KO mice. LPS-instillation also significantly increased the number of pain-like behavior at 24 hours after instillation in WT mice, but not in TRPA1-KO mice. Cystometry 24 hours after LPS-instillation revealed shorter inter-contraction intervals in the WT mice compared with TRPA1-KO mice. In contrast, inflammatory cell infiltration in the bladder suburothelial layer was not significantly different between the two groups. These results indicate that TRPA1 channels are involved in bladder mechanosensory and nociceptive hypersensitivity accompanied with inflammation but not in physiological bladder function or development of bladder inflammation.

## Introduction

Transient receptor potential ankyrin 1 (TRPA1) is a nonselective cation channel expressed primarily by sensory neurons. TRPA1 channel is involved in mechanosensation and chemical nociception activated by exogenous irritants and noxious stimulation^1–4^. Additionally, several studies demonstrated that pharmacological inhibition of TRPA1 with HC-030031, a selective TRPA1 antagonist, attenuated inflammatory- and neuropathy-induced mechanical hypersensitivity in rodents. Similar observations in TRPA1-knock out (KO) mice suggest pathophysiological roles for TRPA1 channel in mechanical or thermal hypersensitivity^5–8^.

Immunohistochemical studies in lower urinary tract demonstrated that TRPA1 was expressed abundantly in the urothelium and C-fiber afferents of rat bladder (9) as well as in the mucosa of human bladder^9,10^. *TRPA1* mRNA expression in bladder tissue with Hunner-type interstitial cystitis, which is characterized by hypersensitive bladder symptoms and extensive bladder inflammation, was upregulated^11–13^. The proportion of bladder afferent neurons expressing functional TRPA1 in L5–S1 dorsal root ganglia (DRGs) was increased, and the selective TRPA1 antagonist HC-030031 attenuated bladder pain-like behavior in mouse models of chemical cystitis^14,15^. These results suggested that TRPA1 might be associated with inflammatory bladder hypersensitivity. In contrast, a study reported that instillation of allyl isothiocyanate, a selective TRPA1 agonist, into rat bladder did not change any of the cystometric parameters^16^. Another study demonstrated no functional changes in urethane-anesthetized cystometry in TRPA1-KO mice^17^.

In this study, we explored *in vitro* and *in vivo* bladder functional phenotypes of TRPA1-KO mice to understand the physiological role of TRPA1 in regulating bladder function. We also compared changes in bladder histology, mechanosensation, and nociception induced by intravesical instillation of lipopolysaccharide (LPS) between wild type (WT) and TRPA1-KO mice to reveal the role of TRPA1 in inflammation and mechanosensory and nociceptive bladder hypersensitivity due to inflammation.

## Results

### Phenotypic bladder function of TRPA1-KO mice in the absence of LPS instillation

#### Frequency/volume measurements

There were no significant differences in body weight between WT and TRPA1-KO mice subjected to frequency/volume measurements for either sex (male, 24.4 ± 0.5 g vs. 23.3 ± 0.4 g, female, 19.4 ± 0.2 g vs. 19.0 ± 0.4 g, respectively; n = 10–22 per group). There were also no significant differences in any of the frequency/volume parameters between the two groups in either sex (Table 1). Mean flow rate was significantly higher in female mice in both WT and TRPA1-KO mice (Supplementary Table S1).

**Table 1:** Frequency volume measurement parameters in WT and TRPA1-KO mice.

	Male			Female		
	WT (n = 10)	TRPA1-KO (n = 10)	*p* value	WT (n = 22)	TRPA1-KO (n = 17)	*p* value
Voiding frequency (times)	18.5 ± 1.72	16.1 ± 1.35	0.14	21.0 ± 1.30	18.0 ± 1.39	0.13
Total voided volume (ml)	2.49 ± 0.10	2.13 ± 0.17	0.09	2.32 ± 0.17	2.04 ± 0.19	0.29
Mean voided volume (μl)	136.7 ± 7.9	135.2 ± 7.6	0.89	115.8 ± 9.9	120.2 ± 11.2	0.77
Mean flow rate (μl/sec)	43.6 ± 1.5	40.7 ± 1.8	0.24	55.1 ± 3.0	56.4 ± 2.8	0.75
Water intake (ml)	4.99 ± 0.39	4.44 ± 0.33	0.32	4.24 ± 0.30	3.94 ± 0.34	0.52

WT, wild-type; TRPA1-KO, Transient receptor potential ankyrin 1-knockout.

No significant differences between WT and TRPA1-KO mice of same sex (unpaired Student’s *t* test).

#### Decerebrated unanesthetized cystometry (CMG) measurements

There were no significant differences in any of the CMG parameters between WT and TRPA1-KO mice in either sex (n = 8–15 per group; Table 2). Inter-contraction interval in TRPA1-KO mice and mean voided volume in both WT and TRPA1-KO mice were significantly greater in female compared to male mice (Supplementary Table S1).

**Table 2:** Parameters of decerebrated unanesthetized cystometry measurements in WT and TRPA1-KO mice.

	Male			Female		
	WT (n = 8)	TRPA1-KO (n = 9)	*p* value	WT (n = 15)	TRPA1-KO (n = 9)	*p* value
Basal pressure (cmH_2_O)	1.41 ± 0.38	1.27 ± 0.30	0.78	1.42 ± 0.25	1.24 ± 0.26	0.52
Threshold pressure (cmH_2_O)	5.04 ± 0.33	4.64 ± 0.43	0.48	5.93 ± 0.33	6.20 ± 0.86	0.65
Maximum pressure (cmH_2_O)	21.09 ± 1.83	24.84 ± 2.07	0.20	27.17 ± 1.62	28.09 ± 1.13	0.91
Inter-contraction interval (sec)	457.0 ± 70.4	480.7 ± 43.4	0.77	610.8 ± 44.1	698.1 ± 46.0	0.42
Mean voided volume (μl)	79.1 ± 10.9	86.6 ± 6.8	0.54	108.7 ± 7.5	125.7 ± 10.0	0.26
Residual volume (μl)	4.9 ± 1.9	4.0 ± 1.3	0.67	2.8 ± 0.7	3.6 ± 1.9	0.26

WT, wild-type; TRPA1-KO, Transient receptor potential ankyrin 1-knockout.

No significant differences between WT and TRPA1-KO mice of same sex (unpaired t-test).

#### *In vitro* functional assessment of detrusor strips

**Figure 1:**
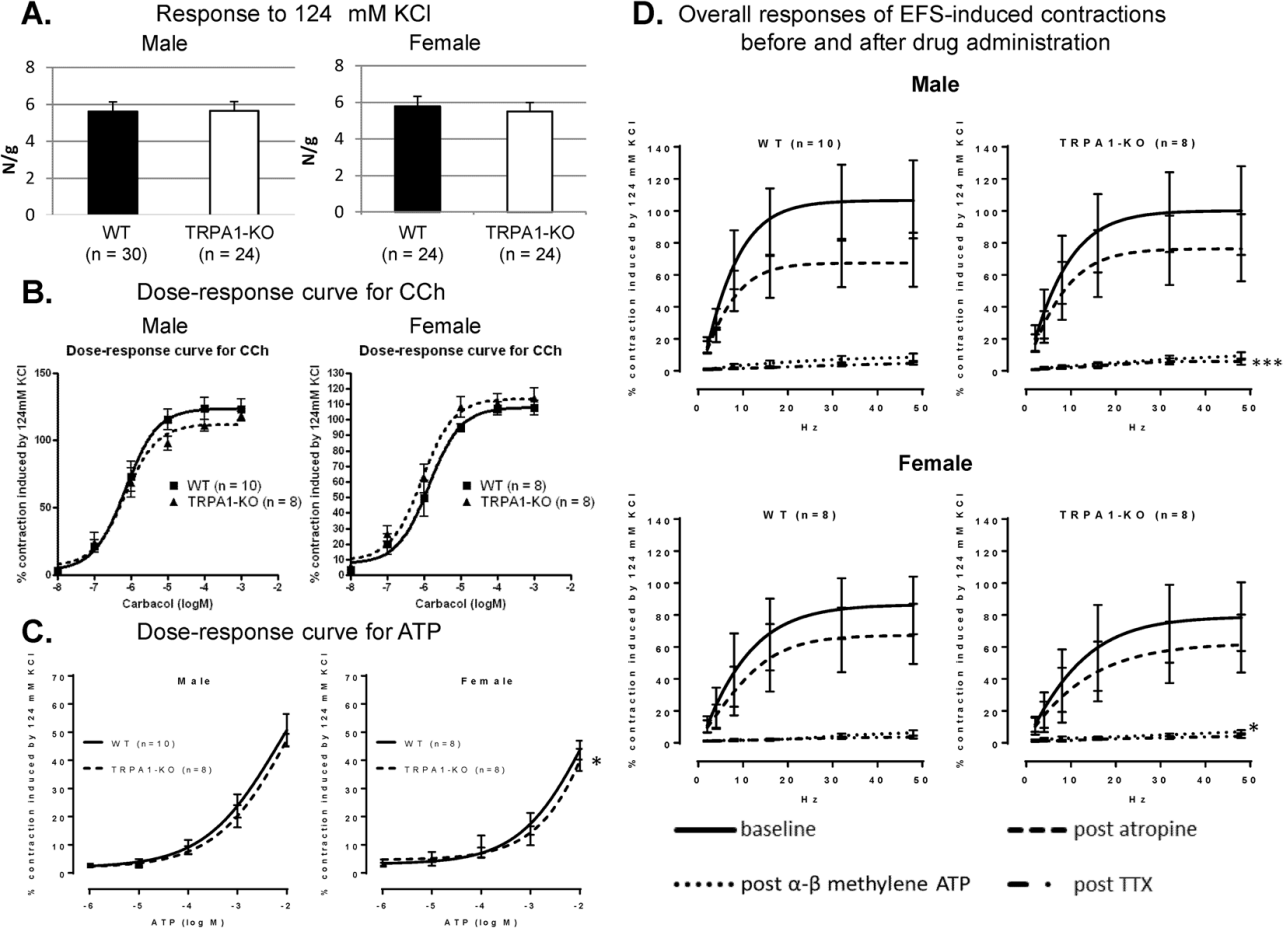
Contractile responses to high K^+^ (**A**), carbachol (CCh, **B**), adenosine triphosphate (ATP, **C**), electric field stimulation (EFS, **D**). Values are expressed as means ± standard error of the mean (SEM). ^*^p < 0.05, ^***^p < 0.001 from WT mice of same sax by f-test of nonlinear regression.

There were no significant differences in the contractile responses to high K^+^ (124 mM KCl) (n = 24–30 from n = 8–10 per group), or carbachol (CCh, 10^−8^–10^−3^ M) (n = 8–10 from n = 8–10 per group) in either sex, or adenosine triphosphate (ATP,: 10^−6^–10^−2^ M) in male mice (n = 8–10 from n = 8–10 per group) between WT and TRPA1-KO mice (Fig. 1A–C). In contrast, the contractile responses to ATP in female TRPA1-KO mice were significantly weaker than those in female WT mice (n = 8 from n = 8 per group, Fig. 1C). There were no significant differences in the amplitude of contractions induced by electric field stimulation (EFS) at any frequency in either sex (n = 8–10 from n = 8–10 per group). Even after atropine administration, no significant differences were detected between the two groups in either sex.

In contrast, after repeated α, β-methylene-ATP (mATP) administration in female mice and tetrodotoxin (TTX) administration in male mice, the amplitude of contractions in TRPA1-KO mice were significantly smaller than that in WT mice in the same sex, although mATP and TTX administration markedly reduced contractility relative to baseline regardless of sex or genotype (Fig. 1D, Supplementary Fig. S1).

### Inflammatory and nociceptive responses to LPS instillation

Because no phenotypic differences in bladder function were observed between WT and TRPA1-KO mice in either sex, inflammatory and nociceptive responses to LPS instillation were evaluated only in female mice.

#### Frequency/volume measurements

**Figure 2:**
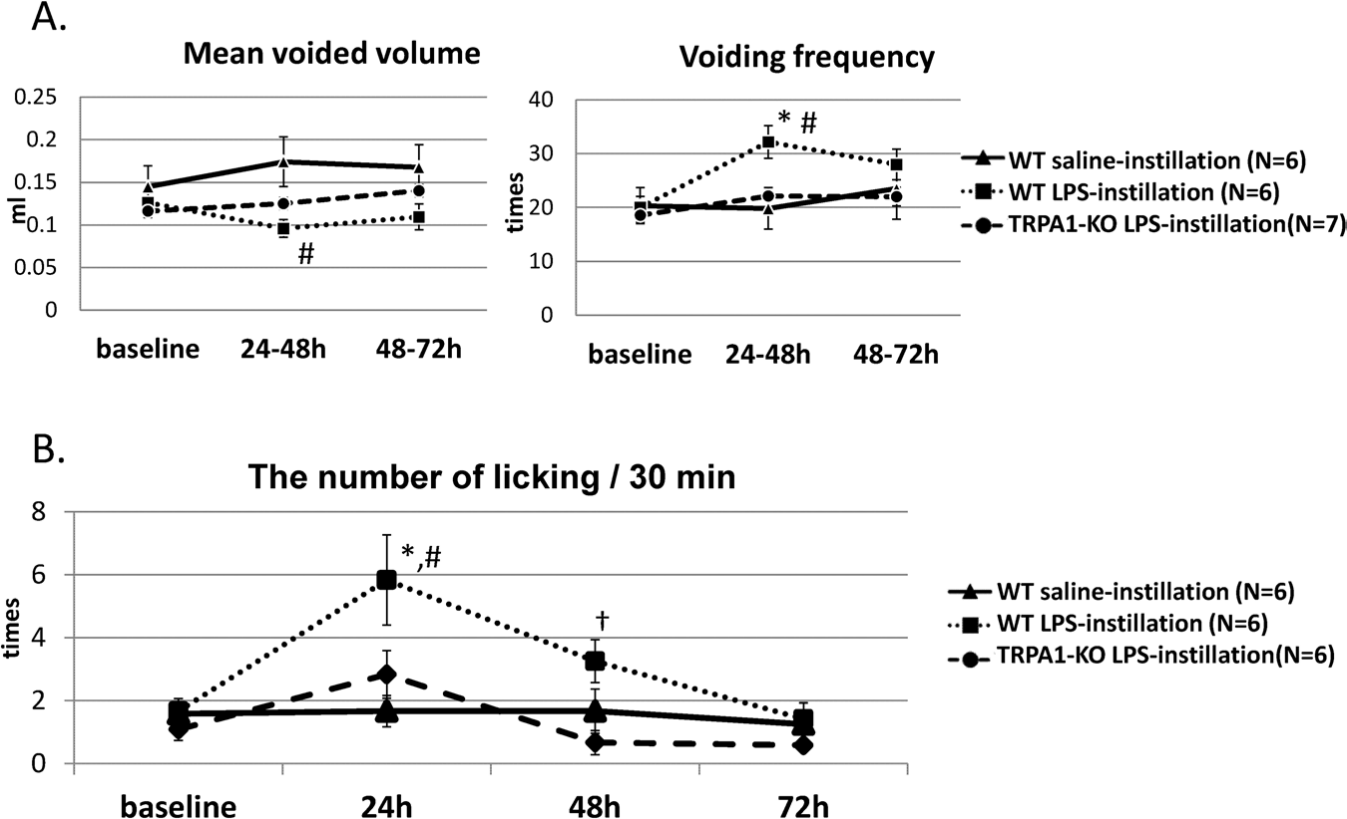
Changes over time in voiding (**A**) and bladder pain-like behavior (**B**) before and after saline or LPS instillation in female WT and TRPA1-KO mice. Values are expressed as mean ± SEM. ^*^p < 0.05 from baseline by Dunnett’s test; ^#^*p* < 0.05 from WT mice with saline instillation by Tukey’s test; ^†^*p < *0.05 from TRPA1-KO mice with LPS instillation by Tukey’s test.

There were no significant differences in voiding frequency or voided volume between the groups at baseline. In WT mice, LPS instillation led a significant increase in voiding frequency and a significant decrease in mean voided volume at 24–48 hours after instillation compared with the saline instillation (n = 6 per group). In contrast, in TRPA1-KO mice, there were no significant changes in either voiding frequency or mean voided volume after LPS instillation compared with the baseline (n = 7; Fig. 2A).

#### Bladder pain-like licking behavior

WT mice with LPS instillation exhibited a significant increase in the number of licking behavior at 24 hours after instillation compared with the baseline and the WT mice with saline instillation. In contrast, TRPA1-KO mice did not exhibit significant changes in the number of licking behavior compared with baseline at 24 hours after LPS instillation and significantly less behavior than the WT mice at 48 hours after LPS instillation (n = 6 per group; Fig. 2B).

#### Decerebrated unanesthetized CMG measurements

**Figure 3:**
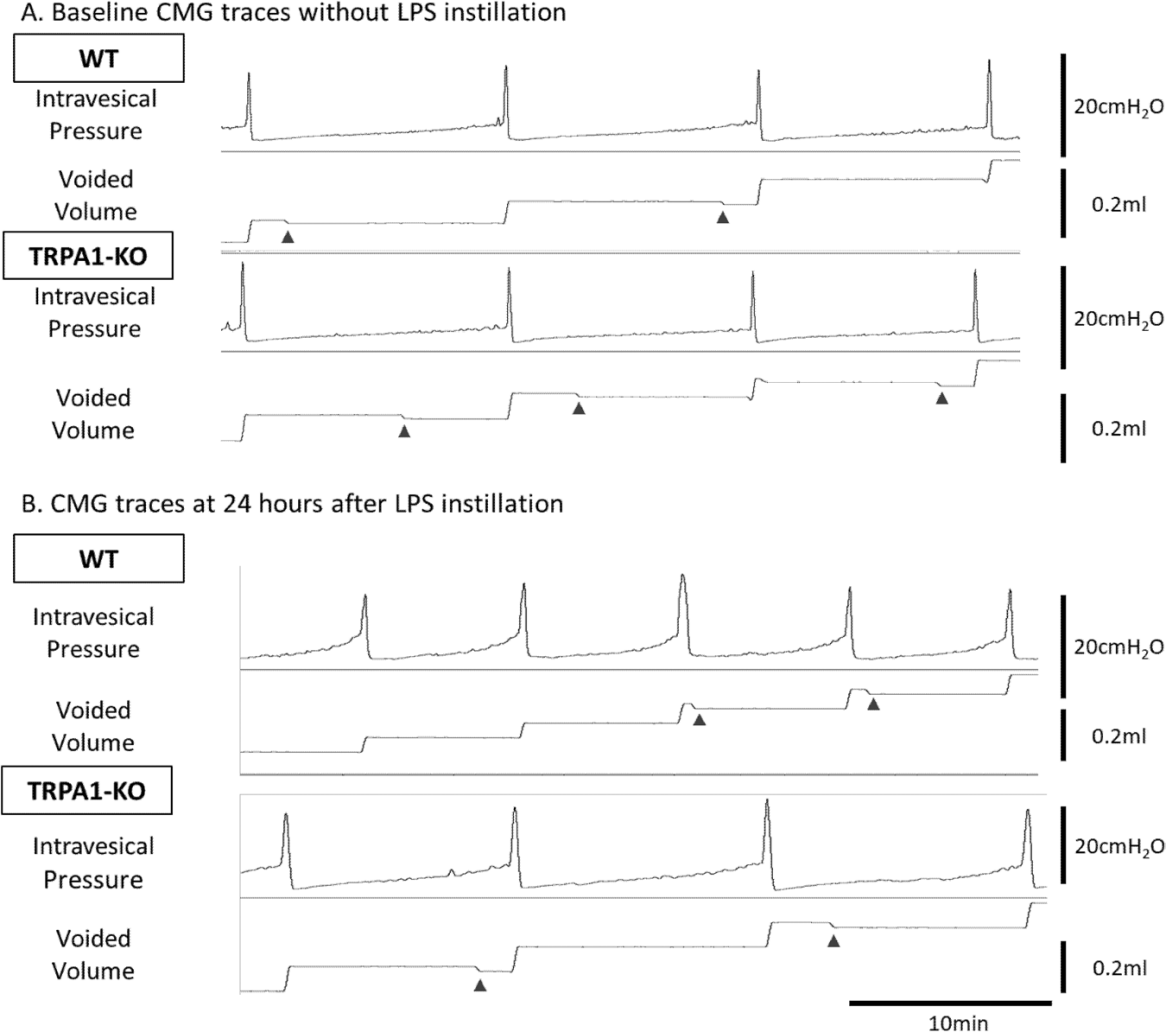
Representative traces of intravesical pressure and voided volume during CMG with continuous saline instillation (10 µl/min) without LPS instillation (baseline) (**A**) and at 24 hours after LPS instillation in decerebrate, unanesthetized female WT and TRPA1-KO mice (**B**). Arrowheads: Mechanical artifacts on the electronic scale.

CMG measurements performed at 24 hours after LPS instillation revealed that interaction between LPS-instillation and genetically TRPA1-KO in inter-contraction interval and voided volume. Moreover, LPS-instilled WT mice exhibited significantly shorter inter-contraction intervals and lower mean voided volume than their baseline data. In contrast, LPS-instilled TRPA1-KO mice did not demonstrate any significant changes compared with their baseline data before LPS-instillation. TRPA1-KO mice showed significantly longer inter-contraction intervals and higher mean voided volumes than the LPS-instilled WT mice (n = 7 per group; Table 3 and Fig. 3).

**Table 3:** Parameters of decerebrated unanesthetized CMG measurements in female WT and TRPA1-KO mice at 24 hours after LPS instillation and comparison to those without LPS instillation (baseline).

	WT	TRPA1-KO	*p* value			
	(n = 7)	(n = 7)	interaction	baseline and post LPS instillation in WT	baseline and post LPS instillation in TRPA1-KO	WT and TRPA1-KO with LPS instillation
Basal pressure (cm H_2_O)	2.61 ± 0.56	1.92 ± 0.31	0.46	0.085	0.11	0.29
Threshold pressure (cm H_2_O)	8.93 ± 0.98	7.27 ± 0.43	0.15	0.061	0.33	0.16
Maximum pressure (cm H_2_O)	24.65 ± 0.99	28.60 ± 2.32	0.40	0.20	0.84	0.14
Inter-contraction interval (sec)	494.6 ± 62.0	768.4 ± 70.2	0.031*	0.043^†^	0.40	0.007^‡‡^
Mean voided volume (μl)	88.0 ± 13.0	128.8 ± 9.2	0.043*	0.047^†^	0.83	0.025^‡^
Residual volume (μl)	3.6 ± 1.2	7.4 ± 3.3	0.39	0.60	0.32	0.30

CMG, cystometry; WT, wild-type; TRPA1-KO, Transient receptor potential ankyrin 1-knockout; LPS, lipopolysaccharide.

^*^p < 0.05: significant interactions between genotype and LPS-instillation (two-way ANOVA test).

^†^p < 0.05: significant differences from same genotype mice at baseline measurements (unpaired Student’s *t* test).

^‡^p < 0.05, ^‡‡^p < 0.01: significant differences from WT mice at 24 hours after LPS instillation (unpaired Student’s *t* test).

### Histological evaluation

**Figure 4:**
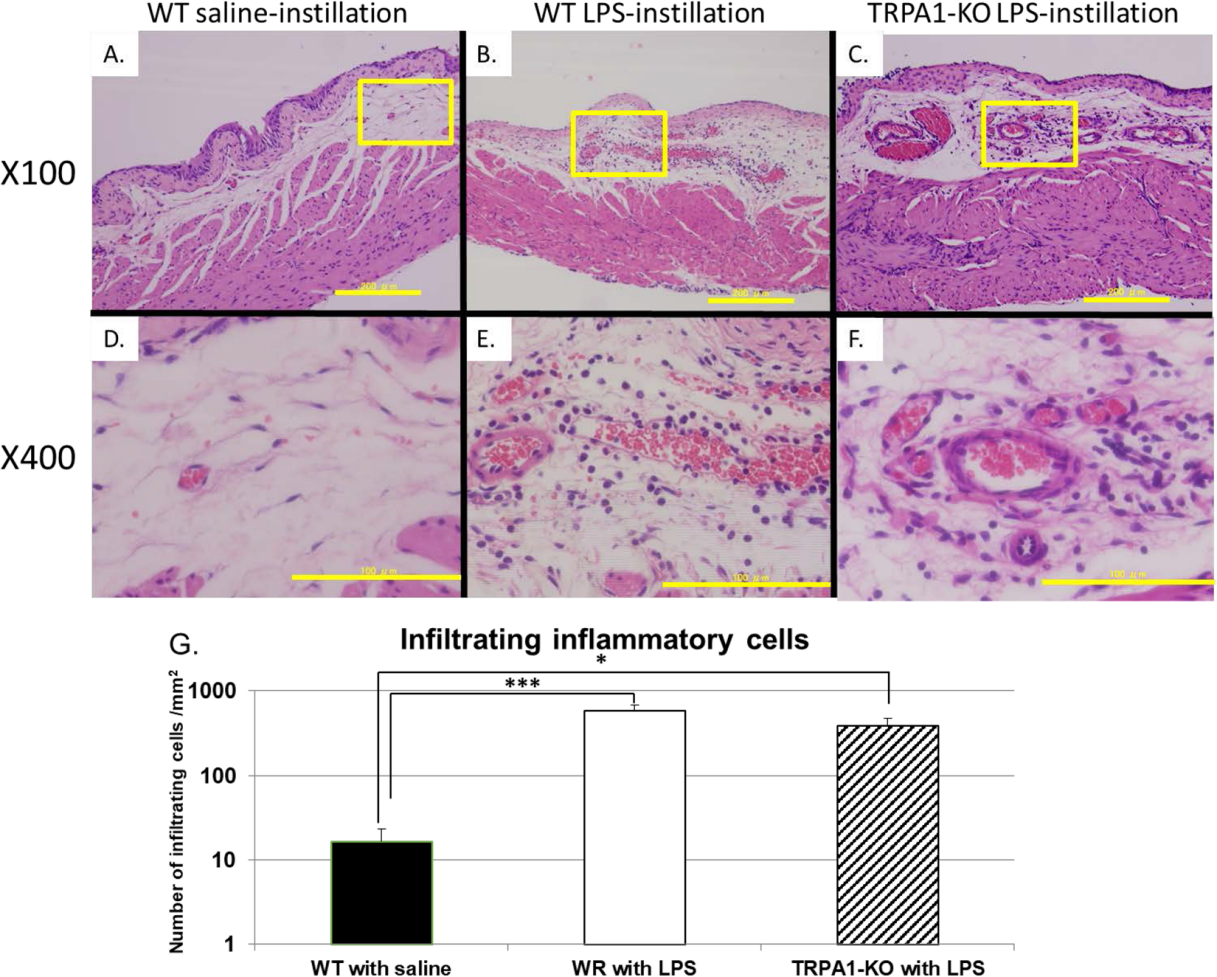
Representative histological images of bladder specimens obtained from saline-instilled (**A**,**D**) or LPS-instilled (**B**,**E**) WT mice and LPS-instilled TRPA1-KO mice (**C**,**F**) at 24 hours after instillation and comparison of the number of infiltrating inflammatory cells in the submucosal layer of the bladder between the groups (n = 7 per group) (**G**). A–C: Scale bar, 200 µm. D–F: Scale bar, 100 µm. The square in each upper panel corresponds to the panel below. ^*^p < 0.05, ^***^p < 0.001 between WT mice with saline instillation by Tukey’s test. There are no significant differences between the WT and the TRPA1-KO mice with LPS instillation (*p* = 0.24).

Histological evaluation of the bladder specimens at 24 hours after saline or LPS instillation revealed that inflammatory cell infiltration and edema in the suburothelial layer were comparable in the bladders of both WT and TRPA1-KO mice received LPS instillation (Fig. 4B,C,E,F). In contrast, neither inflammatory cell infiltration nor edema was observed in the bladder of saline-instilled WT mice (Fig. 4A,D). Compared to those of saline-instilled WT mice, the number of infiltrating inflammatory cells was significantly higher in the bladders of LPS-instilled WT and TRPA1-KO mice (*p* = 0.0009 and 0.034, respectively), which was not significantly different between the WT and TRPA1-KO mice receiving LPS instillation (*p* = 0.24; n = 7 per group; Fig. 4G).

### mRNA expression of TRP channels in bladder and DRG

*TRPA1* mRNA was not expressed in the bladders or the L6 DRGs of TRPA1-KO mice.

**Figure 5:**
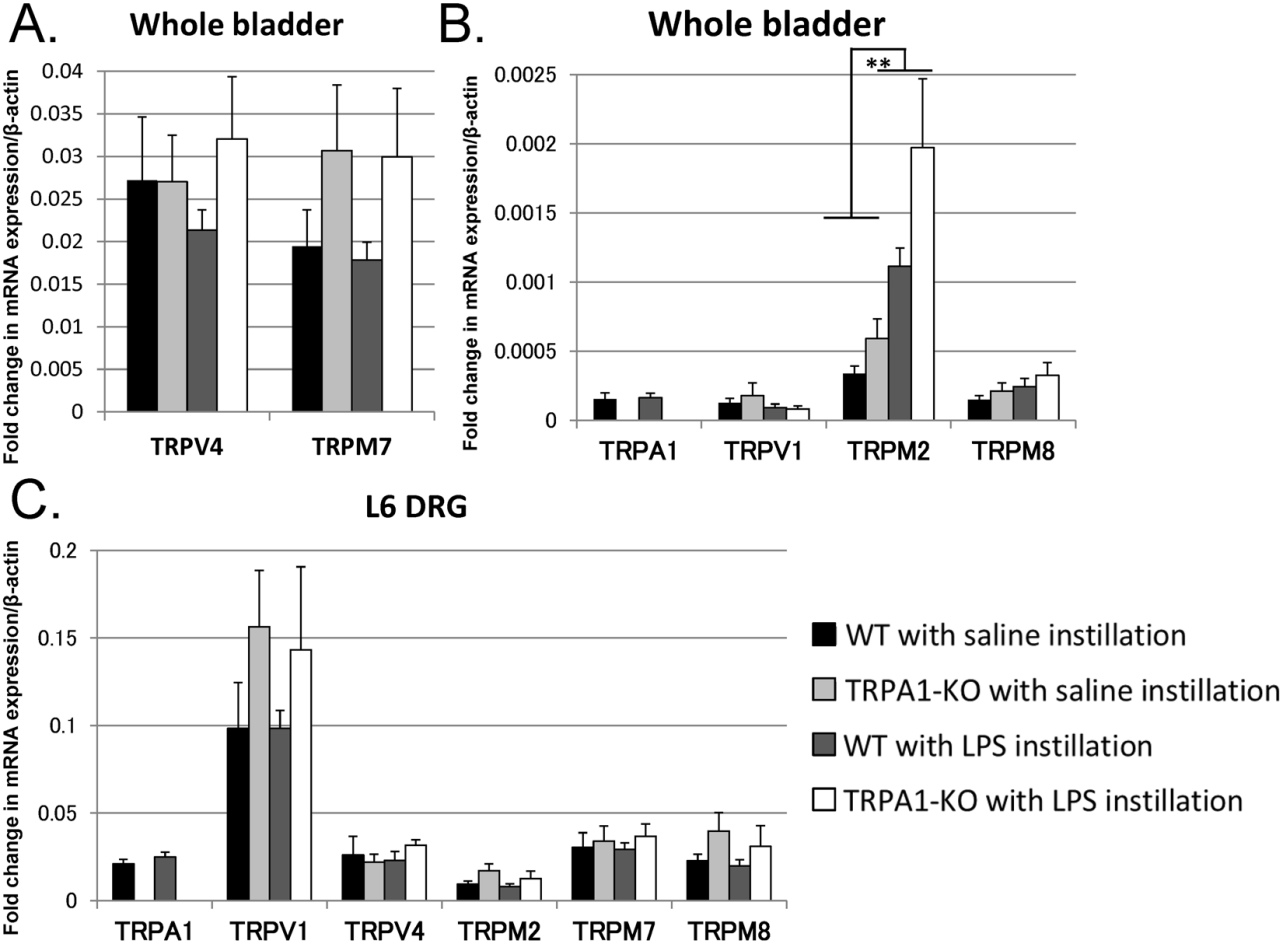
mRNA expression levels of TRP channels in the bladder (**A**,**B**) and those in the L6 dorsal root ganglia (DRGs) (**C**) at 24 hours after saline- or LPS instillation in female WT and TRPA1-KO mice. No interaction effects were significant. ^**^p < 0.01 between the saline instillation and LPS instillation of the bladder (two-way ANOVA test) (**A**,**B**). There were no significant differences in the L6 DRGs between the saline instillation and LPS instillation or between WT and TRPA1-KO mice (**C**).

Two-way ANOVA test revealed that there were no significant interaction effects between LPS instillation and genetic deletion of *TRPA1* in the mRNA expression changes of any TRP channels. The expression of other TRP channels in the bladders or L6 DRGs were not significantly different between the WT and TRPA1-KO mice (n = 5 per group; Fig. 5A–C). LPS instillation significantly increased *TRPM2* expression in the bladders compared to those with saline instillation (Fig. 5B), which was not observed in the L6 DRGs (Fig. 5C). LPS instillation did not affect the expressions of any of the other TRP channels significantly in the bladder or L6 DRGs.

## Discussions

This study revealed that baseline bladder functions in TRPA1-KO mice were similar to those of WT mice and that TRPA1-KO mice exhibited less hypersensitive responses to LPS instillation, with histological inflammatory changes indistinguishable from those of WT mice. These results suggested that TRPA1 channels play minimal roles in physiological control of bladder function or development of LPS-induced bladder inflammation, while contributing to inflammation-induced bladder hypersensitivity in mice.

*In vitro* functional analysis of detrusor strips demonstrated that there were no significant differences in the contractile responses to high K^+^, CCh and EFS before drug administration and after atropine administration between WT and TRPA1-KO mice in either sex. These results suggested that intrinsic and cholinergic contractile property of detrusor smooth muscle were not different between WT and TRPA1-KO mice. Although female TRPA1-KO mice showed significantly lower contractile responses to ATP, this difference in the purinergic contractile property between WT and TRPA1-KO mice may not influence total contractile function.

A previous CMG study in conscious free-moving rats revealed no effect on any CMG parameters with application of a TRPA1 antagonist^16^, and another CMG study showed that none of the CMG parameters in urethane-anesthetized TRPA1-KO mice were different from those of the WT mice. We detected no significant differences in *in vivo* measurements of frequency/volume and CMG between the WT and TRPA1-KO mice in either sex. These findings indicate that TRPA1 channels are unlikely to contribute to the physiological control of the bladder.

We then examined whether TRPA1 channels were involved in the development of bladder inflammation and inflammation-associated hypersensitive responses induced by intravesical instillation of LPS. LPS instillation in WT mice significantly increased the number of bladder pain-like behavioral events at 24 hours after instillation. LPS instillation additionally led an increase in voiding frequency and a decrease in mean voided volume at 24–48 hours after instillation in WT mice. In contrast, TRPA1-KO mice showed weaker responses as determined by bladder pain-like behavior and frequent voiding provoked by LPS instillation at 24–48 hours after instillation. CMG measurements showed longer inter-contraction intervals and higher mean voided volumes in TRPA1-KO mice than those in WT mice, supporting the results of the frequency/volume measurements. Nevertheless, histological evaluation of inflammatory response to LPS instillation revealed that the infiltrating inflammatory cell numbers were similar between the WT and TRPA1-KO mice. Taken together, these results suggested that TRPA1 channels may not contribute to inflammation itself but were involved in increased nociception and mechanosensory responses induced by bladder inflammation. A previous study demonstrated that LPS directly activates TRPA1, which is involved in immediate nociceptive responses to LPS, independently of Tall -like receptor 4^18^. We cannot deny that the direct activation of TRPA1 by LPS contributed to the differences of the differences of nociceptive and micturition behaviors, even though our study evaluated the behavioral changes at 24 hours or later after LPS stimulation but not immediately after its instillation. Further studies evaluating the differences of immediate responses to LPS between WT and TRPA1-KO mice may be needed.

Previous rodent studies reported that TRPA1 antagonists attenuated bladder hyperalgesia and bladder hyperreflexia induced by cyclophosphamide or ifosfamide in inflammatory cystitis models^14,15,19^, which support the findings of the present study utilizing genetic deletion of *TRPA1*. As TRPA1-KO mice exhibited no detectable changes in baseline bladder functions, we speculated that there might be a compensatory increase in the expression of other TRP channels in TRPA1-KO mice. Previous studies, however, demonstrated that mRNA expression levels of *TRPV1* and *TRPM8* were not different between the TRPA1-KO and WT mice^20,21^. In the present study, mRNA expression levels of several TRP channels that could possibly be associated with bladder function, including *TRPV1*, *TRPV4*, *TRPM2*, *TRPM7*, and *TRPM8*, did not change in the bladder or the L6 DRGs in TRPA1-KO mice.

Given that bladder hypersensitivity-associated behaviors were induced after the instillation, we expected an increase in expression of TRPA1channels in WT mice after LPS instillation. However, mRNA expression of *TRPA1* did not change in the bladder or the L6 DRGs at least at 24 hours after LPS instillation. DeBerry *et al*. reported that the mRNA expression level of *TRPA1* in L6–S1 DRGs of mice did not change in response to repeated cyclophosphamide administration^14^; however, in the same cystitis model, they also observed an upregulation of *TRPA1* mRNA expression by single-cell reverse transcription-polymerase chain reaction (RT-PCR) of neuronal cells in L5–S1 DRGs innervating the bladder^22^. A more detailed examination such as evaluation by single-cell RT-PCR of the neuronal cells in L6 DRGs innervating the bladder is needed to understand the precise roles of TRPA1 in DRG.

Interestingly, we found that the *TRPM2* mRNA expression was upregulated at 24 hours after LPS instillation in both the WT and TRPA1-KO bladders. TRPM2 channels were reported to be expressed abundantly in immune cells including monocytes, macrophages, and neutrophils, contributing to inflammatory and neuropathic pain^23,24^. Thus, our observation regarding *TRPM2* mRNA expression is consistent with those reports, with concomitant histological inflammatory changes in both mouse lines. The contribution of TRPM2 channel to physiological and pathophysiological bladder functions require further investigation.

Our study has several limitations. First, potential compensatory changes in bladder function due to genetic deletion of *TRPA1*, which could mask the actual functional roles of TRPA1 cannot be excluded. Second, our study lacked the data of the inflammatory and nociceptive responses of TRP1-KO mice with saline instillation. TRP1-KO mice with saline instillation would be expected to show the similar results to WT mice with saline instillation, because there were little phenotypic differences in physiological condition between WT and TRPA1-KO mice. However, comparing 4 groups including TRP1-KO mice with saline instillation may be more suitable. Moreover, we evaluated changes by cystometry, histology, and RT-PCR only at 24 hours after LPS instillation. Evaluation at multiple time points is needed for precise determination of differences between WT and TRPA1-KO mice. Third, the degree of inflammatory changes in bladder tissue was quantified only by counting the number of infiltrating inflammatory cells by hematoxylin-eosin (H-E) staining and the types of inflammatory cells were not identified. Immunohistochemistry staining and digital image analysis could evaluate quantitative evaluation of each inflammatory cells in the bladder tissue more precisely, which may help to reveal the differences of inflammatory changes between WT and TRAP1-KO mice^13,25^. Finally, we did not conduct direct evaluations of bladder afferent nerve activity to assess bladder sensory function or of elevation of the oxdative stress markers such as hydrogen peroxide in the bladder tissue with LPS-induced inflammation. Further investigations including these evaluations are needed.

In conclusion, this is the first study demonstrating that TRPA1 channels contribute to inflammation-associated bladder mechanosensory and nociceptive hypersensitivity using TRPA1-KO mice. These findings suggest TRPA1 channels as promising pharmacological target for the treatment of inflammatory hypersensitive bladder disorders.

## Methods

### Animals

A total of 19 male and 42 female TRPA1-KO mice and 18 male and 46 female WT littermates (10–12-weeks old) were used. Mice were maintained under standard laboratory conditions with a 12-h light (9:00 a.m. to 9:00 p.m.) and 12-h dark (9:00 p.m. to 9:00 a.m.) cycles with free access to food and water. Experimental protocols were approved by the animal ethics committee of the University of Tokyo Graduate School of Medicine in Tokyo, Japan, and were in line with the NIH guidelines for the care and use of experimental animals. TRPA1-KO mice were generated as reported previously^26^. The TRPA1-KO mouse line obtained from Jackson Laboratory (Bar Harbor, ME) was backcrossed with C57BL/6 J mice for at least ten generations to eliminate any background effects on the phenotypes.

### Induction of cystitis

Under isoflurane anesthesia, a polyethylene catheter (PE-10; Clay Adams, Parsippany, NJ, USA) was inserted transurethrally into the bladder. After draining urine, 100 µL saline containing 2.0 mg/ml LPS extracted from *Escherichia coli* O55:B5 was instilled intravesically for one hour. As a control, vehicle (saline) was instilled instead of LPS. During the 1-hour instillation period, LPS or saline was exchanged to a new solution at 30 minutes after instillation to avoid dilution with urine. After instillation, the solution was drained, and the mouse was allowed to recover from anesthesia. LPS dose was determined based on a previous study^27^ and our pilot investigation.

### Frequency/volume measurements

Each conscious mouse was separately placed in an MCM/TOA-UF001-006 metabolic cage (Mitsubishi Chemical Medience, Tokyo, Japan) without any restraint, as reported previously^28–30^. After a 24-hour adaptation period, voided volume, voiding frequency, mean flow rate, and water intake volume were continuously recorded for 24 hours with a PowerLab (AD Instruments, Sydney, Australia) data acquisition system. All mice had free access to water and food during recordings.

For evaluation of responses to LPS, after baseline measurements of voiding behavior for 24 hours, the mice received intravesical LPS or saline instillation and were placed back into the cage. After a 24-hour adaptation period, voiding behavior was monitored for another 48 hours.

### Bladder pain-like nociceptive behavior

Assessment of bladder pain-like behavior was performed as described previously, with minor modifications^31,32^. Each mouse was placed under a lucent plastic box and habituated to the environment for one hour. Next, bladder pain-like behavior such as licking or biting of the skin of the lower abdomen close to the bladder was monitored on a video-camera (HDR-XR500V, Sony Corporation, Tokyo, Japan) for the next 30 minutes as baseline^33^. Thereafter, the mice received intravesical LPS or saline instillation, and bladder pain-like behavior was monitored again for 30 minutes at 24, 48, and 72 hours after instillation. Number of behaviors for each mouse was counted by two investigators in a blinded manner, and the values were averaged.

### Decerebrated unanesthetized CMG measurements

CMG measurements were performed in decerebrate, unanesthetized mice according to the previously reported methods (20). In brief, under isoflurane anesthesia, a PE-50 catheter (Clay Adams) was implanted into the bladder through the dome. Skin from the scalp was removed, and skull was opened, and decerebration was performed at pre-collicular position. To eliminate the potential influence of anesthesia, more than two hours after surgery, the intravesical catheter was connected via a three-way stopcock to a pressure transducer (DX-100, Nihon Kohden, Tokyo, Japan) and a syringe pump (KDS 200, Muromachi Kikai, Tokyo, Japan) for saline instillation at 10 µl/min. Intravesical pressure and voided volume were recorded continuously using a data acquisition program (PowerLab). After stable and reproducible recordings were obtained, micturition cycles during a 1-hour period were averaged, and the following cystometric parameters were analyzed: basal pressure (minimum bladder pressure), threshold pressure (bladder pressure at onset of micturition), maximum pressure (maximum bladder pressure during micturition), voided volume, residual volume (infusion volume–voided volume) and inter-contraction interval (duration between two micturitions)^34^.

### *In vitro* functional studies of detrusor strips

*In vitro* assessment of detrusor muscle function was performed as described in our previous reports^28,30,35^. After mice were sacrificed by pentobarbital sodium overdose, bladder bodies were harvested, and longitudinal, full-thickness detrusor strips were transferred to 5 ml organ baths. After a 2-hour equilibration period with a 10 mN stable tension, functional assessment was initiated. Briefly, the strip was first exposed to a high K^+^-containing (124 mM KCl) Krebs solution. Then, contractile responses to the following stimuli were examined: CCh (10^−8^–10^−3^ M), ATP: 10^−6^–10^−2^ M, electric field stimulation (EFS; pulse width, 0.8 milliseconds, 50 V; pulse duration, 10 seconds; stimulation interval, 2 minutes at 2, 4, 8, 16, 32, and 48 Hz). After baseline EFS measurement, contractions were evoked by EFS again after 10^−6^ M atropine administration, purinoceptor desensitization by repeated administrations of 10^−5^ M mATP, and finally 10^−6^ M TTX.

### Histological examination

After mice were sacrificed by pentobarbital sodium overdose, bladders were harvested and opened at the dorsal side of the bladder neck along the midline toward the dome. Isolated bladder specimens were fixed in 4% paraformaldehyde-phosphate buffered saline, embedded in paraffin, and cut into 3-μm sections. Histological evaluation was performed with hematoxylin-eosin (H-E) staining, and three sections of submucosal layer with maximum infiltration of inflammatory cells per bladder were chosen by a double-blinded evaluator. Thereafter, number of infiltrating inflammatory cells was counted using a counting grid at 400× magnification by two investigators, and the values were averaged.

### Real-time RT-PCR

After mice were sacrificed with pentobarbital sodium overdose, bladders and bilateral L6 DRGs were dissected and immediately placed in RNA*later* RNA stabilization reagent (QIAGEN, Venlo, Netherland). Total RNA extracted from tissues using miRNeasy Mini kit (QIAGEN) was reverse-transcribed to cDNA using SuperScript VILO Master Mix (Life Technologies, Carlsbad, CA, USA) according to the manufacturer’s protocol. For relative quantification of mRNA expression, real-time RT-PCR was performed using Power SYBR Green PCR Master Mix and gene specific primers with the StepOnePlus Real-Time PCR System (Life Technologies). Primer sequences are provided in Supplementary Table S2.

### Drugs

CCh, ATP, atropine, and TTX were purchased from Wako Chemical (Tokyo, Japan). LPS, and mATP were purchased from Sigma-Aldrich (St. Louis, MO, USA). Krebs solution was composed of 118 mM NaCl, 4.7 mM KCl, 2.5 mM CaCl_2_, 25.0 mM NaHCO_3_, 1.2 mM KH_2_PO_4_, and 11 mM glucose (pH 7.4).

### Statistical analysis

All data were expressed as means ± standard error of means. Dose responses for CCh and ATP, and frequency responses for EFS were analyzed by f-test of nonlinear regression, and other results were analyzed using unpaired Student’s *t* test, Tukey’s test, the Mann-Whitney *U* test, repeated measures analysis of variance (ANOVA) followed by Dunnett’s *post hoc* analysis, or two-way ANOVA test. *P* < 0.05 was considered statistically significant.

## Electronic supplementary material

Supplementary Figure S1 and Table S1 and S2

## References

[1] Story Gina M., Peier Andrea M., Reeve Alison J., Eid Samer R., Mosbacher Johannes, Hricik Todd R., Earley Taryn J., Hergarden Anne C., Andersson David A., Hwang Sun Wook, McIntyre Peter, Jegla Tim, Bevan Stuart, Patapoutian Ardem (2003). ANKTM1, a TRP-like Channel Expressed in Nociceptive Neurons, Is Activated by Cold Temperatures. Cell.

[2] Jordt SE (2004). Mustard oils and cannabinoids excite sensory nerve fibres through the TRP channel ANKTM1. Nature.

[3] Kaneko Y, Szallasi A (2014). Transient receptor potential (TRP) channels: a clinical perspective. British journal of pharmacology.

[4] Zygmunt PM, Hogestatt ED (2014). TRPA1. Handb Exp Pharmacol.

[5] Eid SR (2008). HC-030031, a TRPA1 selective antagonist, attenuates inflammatory- and neuropathy-induced mechanical hypersensitivity. Mol Pain.

[6] Dai Y (2007). Sensitization of TRPA1 by PAR2 contributes to the sensation of inflammatory pain. J Clin Invest.

[7] Zhao M (2012). Acute cold hypersensitivity characteristically induced by oxaliplatin is caused by the enhanced responsiveness of TRPA1 in mice. Mol Pain.

[8] Fernandes ES (2011). A distinct role for transient receptor potential ankyrin 1, in addition to transient receptor potential vanilloid 1, in tumor necrosis factor α–induced inflammatory hyperalgesia and Freund’s complete adjuvant–induced monarthritis. Arthritis & Rheumatism.

[9] Du S (2008). Differential expression profile of cold (TRPA1) and cool (TRPM8) receptors in human urogenital organs. Urology.

[10] Streng T (2008). Distribution and function of the hydrogen sulfide-sensitive TRPA1 ion channel in rat urinary bladder. European urology.

[11] Homma Y (2013). Increased mRNA expression of genes involved in pronociceptive inflammatory reactions in bladder tissue of interstitial cystitis. The Journal of urology.

[12] Homma Y (2016). Clinical guidelines for interstitial cystitis and hypersensitive bladder updated in 2015. International journal of urology: official journal of the Japanese Urological Association.

[13] Maeda D (2015). Hunner-Type (Classic) Interstitial Cystitis: A Distinct Inflammatory Disorder Characterized by Pancystitis, with Frequent Expansion of Clonal B-Cells and Epithelial Denudation. PloS one.

[14] DeBerry JJ, Schwartz ES, Davis BM (2014). TRPA1 mediates bladder hyperalgesia in a mouse model of cystitis. Pain.

[15] Pereira LM (2013). Blockade of TRPA1 with HC-030031 attenuates visceral nociception by a mechanism independent of inflammatory resident cells, nitric oxide and the opioid system. European journal of pain (London, England).

[16] Minagawa T, Aizawa N, Igawa Y, Wyndaele JJ (2014). The role of transient receptor potential ankyrin 1 (TRPA1) channel in activation of single unit mechanosensitive bladder afferent activities in the rat. Neurourology and urodynamics.

[17] Uvin P (2015). Essential role of transient receptor potential M8 (TRPM8) in a model of acute cold-induced urinary urgency. European urology.

[18] Meseguer V (2014). TRPA1 channels mediate acute neurogenic inflammation and pain produced by bacterial endotoxins. Nat Commun.

[19] Chen Z, Du S, Kong C, Zhang Z, Mokhtar AD (2016). Intrathecal administration of TRPA1 antagonists attenuate cyclophosphamide-induced cystitis in rats with hyper-reflexia micturition. BMC urology.

[20] Bautista DM (2006). TRPA1 mediates the inflammatory actions of environmental irritants and proalgesic agents. Cell.

[21] Taylor‐Clark TE (2008). Relative contributions of TRPA1 and TRPV1 channels in the activation of vagal bronchopulmonary C‐fibres by the endogenous autacoid 4‐oxononenal. The Journal of Physiology.

[22] DeBerry JJ, Saloman JL, Dragoo BK, Albers KM, Davis BM (2015). Artemin Immunotherapy Is Effective in Preventing and Reversing Cystitis-Induced Bladder Hyperalgesia via TRPA1 Regulation. J Pain.

[23] Faouzi M, Penner R (2014). TRPM2. Handb Exp Pharmacol.

[24] Haraguchi K (2012). TRPM2 contributes to inflammatory and neuropathic pain through the aggravation of pronociceptive inflammatory responses in mice. The Journal of neuroscience: the official journal of the Society for Neuroscience.

[25] Akiyama Y (2016). Increased CXCR3 Expression of Infiltrating Plasma Cells in Hunner Type Interstitial Cystitis. Scientific reports.

[26] Kwan KY (2006). TRPA1 Contributes to Cold, Mechanical, and Chemical Nociception but Is Not Essential for Hair-Cell Transduction. Neuron.

[27] Charrua A (2009). GRC-6211, a new oral specific TRPV1 antagonist, decreases bladder overactivity and noxious bladder input in cystitis animal models. The Journal of urology.

[28] Ito H (2015). Long-term caloric restriction in rats may prevent age related impairment of *in vitro* bladder function. The Journal of urology.

[29] Aizawa N, Homma Y, Igawa Y (2013). Influence of High Fat Diet Feeding for 20 Weeks on Lower Urinary Tract Function in Mice. Lower urinary tract symptoms.

[30] Kamei J (2018). Age-related changes in function and gene expression of the male and female mouse bladder. Scientific reports.

[31] Murakami-Nakayama M (2015). Polaprezinc attenuates cyclophosphamide-induced cystitis and related bladder pain in mice. Journal of pharmacological sciences.

[32] Tanaka Junichi, Yamaguchi Kaoru, Ishikura Hiroyasu, Tsubota Maho, Sekiguchi Fumiko, Seki Yukari, Tsujiuchi Toshifumi, Murai Akira, Umemura Takehiro, Kawabata Atsufumi (2014). Bladder pain relief by HMGB1 neutralization and soluble thrombomodulin in mice with cyclophosphamide-induced cystitis. Neuropharmacology.

[33] Aizawa Naoki, Wakamatsu Daisuke, Kida Jun, Otsuki Takeya, Saito Yasuho, Matsuya Hidekazu, Homma Yukio, Igawa Yasuhiko (2015). Inhibitory effects of retigabine, a Kv7 channel activator, on mechanosensitive primary bladder afferent activities and nociceptive behaviors in rats. Neurourology and Urodynamics.

[34] Andersson KE, Soler R, Fullhase C (2011). Rodent models for urodynamic investigation. Neurourology and urodynamics.

[35] Igawa Yasuhiko, Kumano Shintaro, Aizawa Naoki, Saito Yasuho, Ito Hiroki, Watanabe Shuzo, Takahashi Nobuyuki, Tajimi Masaomi, Nishimatsu Hiroaki, Homma Yukio (2014). Changes in the Function and Expression of T-Type and N-Type Calcium Channels in the Rat Bladder after Bladder Outlet Obstruction. The Journal of Urology.

