# Supplementary material for: Attenuated lipopolysaccharide-induced inflammatory bladder hypersensitivity in mice deficient of transient receptor potential ankilin1
Source: Sci Rep. 2018 Oct 23;8:15622. doi: 10.1038/s41598-018-33967-x (PMC6199359; doi:10.1038/s41598-018-33967-x)
Supplement: Supplementary file 1 — Supplementary Figure S1 and Table S1 and S2 [file 41598_2018_33967_MOESM1_ESM.docx]

**Attenuated lipopolysaccharide-induced inflammatory bladder hypersensitivity in mice deficient of transient receptor potential ankilin1**

Jun Kamei^1,2^, Naoki Aizawa^1^, Takayuki Nakagawa^3,4^, Shuji Kaneko^4^, Haruki Kume^2^,　Yukio Homma^2,5^, and Yasuhiko Igawa^1^

1. Department of Continence Medicine, The University of Tokyo Graduate School of Medicine, Tokyo, Japan

2. Department of Urology, The University of Tokyo Graduate School of Medicine, Tokyo, Japan

3. Department of Clinical Pharmacology and Therapeutics, Kyoto University Hospital, Kyoto, Japan

4. Department of Molecular Pharmacology, Graduate School of Pharmaceutical Sciences, Kyoto University, Kyoto, Japan

5. Department of Urology, Japan Red Cross Medical Center, Tokyo, Japan

**Corresponding author**

Yasuhiko Igawa, MD., PhD.

Professor and Chairman, Department of Continence Medicine, The University of Tokyo Graduate School of Medicine, Tokyo, Japan

TEL & FAX: +81-3-5800-9792

E-mail: [yigawa-jua@umin.ac.jp](mailto:yigawa-jua@umin.ac.jp)

**Supplementary Table S1.** Comparing the sex differences of the parameters of frequency volume and decerebrated unanesthetized cystometry measurements in WT and TRPA1-KO mice

|  |  | WT | | | TRPA1-KO | | | |
| --- | --- | --- | --- | --- | --- | --- | --- | --- |
|  |  | Male | Female | *p* value |  | Male | Female | *p* value |
| Frequency volume measurements |  | (n = 10) | (n = 22) |  |  | (n = 10) | (n = 17) |  |
| Voiding frequency (times) |  | 18.5 ± 1.72 | 21.0 ± 1.30 | 0.11 |  | 16.1 ± 1.35 | 18.0 ± 1.39 | 0.37 |
| Total voided volume (ml) |  | 2.49 ± 0.10 | 2.32 ± 0.17 | 0.11 |  | 2.13 ± 0.17 | 2.04 ± 0.19 | 0.28 |
| Mean voided volume (μl) |  | 136.7 ± 7.9 | 115.8 ± 9.9 | 0.40 |  | 135.2 ± 7.6 | 120.2 ± 11.2 | 0.75 |
| Mean flow rate (μl/sec) |  | 43.6 ± 1.5 | 55.1 ± 3.0 | 0.002** |  | 40.7 ± 1.8 | 56.4 ± 2.8 | 0.0005*** |
| Water intake (ml) |  | 4.99 ± 0.39 | 4.24 ± 0.30 | 0.17 |  | 4.44 ± 0.33 | 3.94 ± 0.34 | 0.34 |
| Cystometry measurements |  | (n = 8) | (n = 15) |  |  | (n = 9) | (n = 9) |  |
| Basal pressure (cmH_2_O) |  | 1.41 ± 0.38 | 1.42 ± 0.25 | 0.99 |  | 1.27 ± 0.30 | 1.24 ± 0.26 | 0.93 |
| Threshold pressure (cmH_2_O) |  | 5.04 ± 0.33 | 5.93 ± 0.33 | 0.10 |  | 4.64 ± 0.43 | 6.20 ± 0.86 | 0.13 |
| Maximum pressure (cmH_2_O) |  | 21.09 ± 1.83 | 27.17 ± 1.62 | 0.078 |  | 24.84 ± 2.07 | 28.09 ± 1.13 | 0.19 |
| Inter-contraction interval (sec) |  | 457.0 ± 70.4 | 610.8 ± 44.1 | 0.069 |  | 480.66 ± 43.4 | 698.1 ± 46.0 | 0.003** |
| Mean voided volume (μl) |  | 79.1 ± 10.9 | 108.7 ± 7.5 | 0.034* |  | 86.6 ± 6.8 | 125.7 ± 10.0 | 0.005** |
| Residual volume (μl) |  | 4.9 ± 1.9 | 2.8 ± 0.7 | 0.33 |  | 4.0 ± 1.3 | 3.6 ± 1.9 | 0.89 |

WT, wild-type; TRPA1-KO, Transient receptor potential ankyrin 1-knockout

* *p* < 0.05, ** *p* < 0.01, *** *p* < 0.001: from male mice of same genotype (unpaired Student’s *t* test)

**Supplementary Figure S1.**


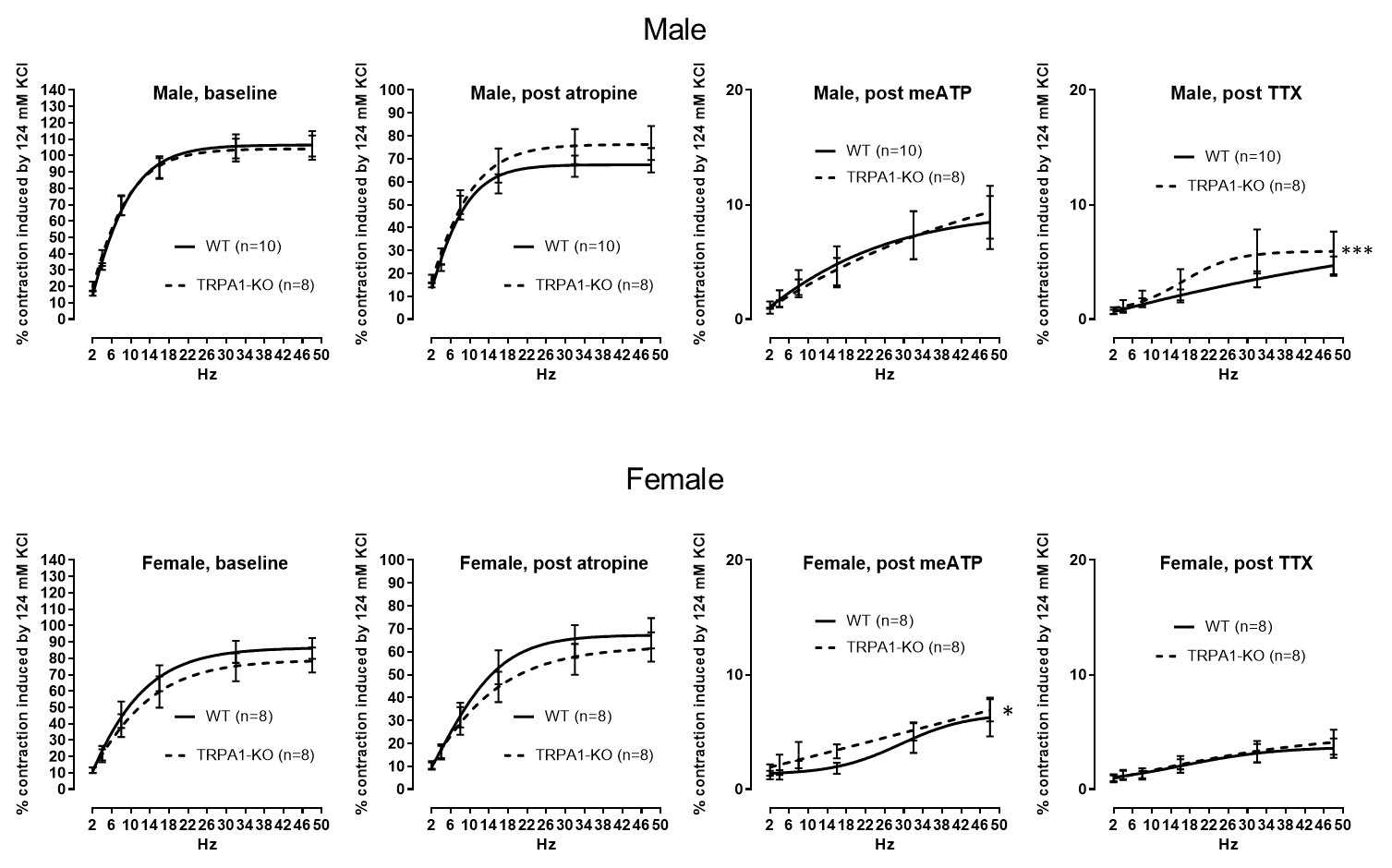


**Supplementary Figure S1. Frequency response curves for EFS in WT and TRPA1-KO mice of both sexes at baseline and after each drug administration.** Values are expressed as means ± standard error of the mean (SEM).

* *p* < 0.05, *** *p* < 0.001 from WT mice of same sax by f-test of nonlinear regression.

**Supplementary Table S2.** List of primers used for real-time reverse transcription-polymerase chain reaction

| Gene | Sequence |
| --- | --- |
| mTRPA1 | 5’- GTCCAGGGCGTTGTCTATCG-3’ |
|  | 5’- CGTGATGCAGAGGACAGAGAT-3’ |
| mTRPM2 | 5’-ACAGACAATGCCTGGATCG-3’ |
|  | 5’-TGGATCATGAGTGTGCAGGT-3’ |
| mTRPM7 | 5’- AGGCATGTTACAAAGCCTGCACTG -3’ |
|  | 5’- AGGCCCTGGGTTTGATCCTCAATA -3’ |
| mTRPM8 | 5’- GGCTCATCCACATTTTCACC -3’ |
|  | 5’- CACCATCCACACAGCAAAGA -3’ |
| mTRPV1 | 5’- CGGAAGACAGATAGCCTGAA -3’ |
|  | 5’- GCTCCATTCTCCACCAAGAG -3’ |
| mTRPV4 | 5’- TGCTGGTCACCTACATCATCCTCA-3’ |
|  | 5’- ACTGCAACTTCCAGATGTGCTTGC -3’ |
| mβ-actin | 5’- GGCTGTATTCCCCTCCATCG -3’ |
|  | 5’- CCAGTTGGTAACAATGCCATGT -3’ |
